# Progressive dyspnoea following the treatment of *Mycobacterium abscessus* infection in an individual with relapsing granulamatosis with polyangitis (Wegener’s), complicated by hearing loss requiring cochlear implantation

**DOI:** 10.1186/1471-2466-12-47

**Published:** 2012-09-04

**Authors:** Senyo K Tagboto, Ajay G Venkatesh

**Affiliations:** 1 Consultant in Internal Medicine & Nephrology Cypress Regional Hospital 2004 Saskatchewan Dr., Swift Current, Saskatchewan, S9H 5M8, Canada; 2 Cypress Regional Hospital 2004 Saskatchewan Dr. Swift Current, Saskatchewan, S9H 5M8, Canada

**Keywords:** Granulomatosis with polyangitis (Wegener’s), Vasculitis, *Mycobacterium abscessus*, Dyspnoea, Pulmonary fibrosis

## Abstract

**Backgound:**

Granulomatosis with polyangitis (Wegener’s) is a vasculitic disease predominantly affecting the lungs, skin, kidneys, ears, nose and throat. *Mycobacterium abscessus* is an uncommon rapidly growing mycobacterium causing sporadic lung disease. This is the first report of both GPA and *Mycobacterium abscessus* pulmonary disease reported in literature.

**Case Presentation:**

We present a case report of a 33 year old Caucasian man with relapsing disease complicated by pulmonary infection with *Mycobacterium abscessus*. He subsequently required bilateral cochlear implantation for progressive sensori-neural hearing loss. His *M. abscessus* was treated successfully with a prolonged course of antimicrobial therapy. His Granulomatosis with polyangitis (Wegener’s) relapsed towards the end of antimicrobial therapy and required treatment. Shortly after completing his antimicrobial therapy and relapse, he developed progressive dyspnea due to pulmonary fibrosis.

**Conclusion:**

The potential causes of his progressive dyspnoea are discussed including the potential role of his underlying disease and treatment.

## Background

Granulomatosis with polyangitis (GPA) is a vasculitic disorder affecting small and medium sized arteries. It commonly presents with ear, nose and throat, pulmonary, skin and renal manifestations. Anti-neutrophil cytoplasmic antibodies (ANCA) are present in 82 – 94% of people with GPA, primarily directed against proteinase 3 (PR3) [1]. The diagnosis may also be made by tissue biopsy.

*Mycobacterium abscessus* is one of a group of rapidly growing mycobacterium (RGM), (including *M. fortuitum, M. chelonae, M. smegmatis, M. mucogenicum, M. neoaurum,* and *M. peregrinum).* They are ubiquitous in nature and can be found in soil, bioaerosols, and water. Several strains have intrinsic resistance to many antibiotics, which complicates treatment. *M. abscessus* is the most pathogenic of this group and occasionally causes pulmonary infection, usually but not invariably in immunocompromised patients or people with underlying lung disease such as cystic fibrosis [2,3]. We could find no reports of this infection complicating GPA.

We report a case of pulmonary *M. abscessus* infection in a 33 year old man with GPA, successfully treated with amikacin, clarythromycin and cefoxitin. His sensori-neural hearing loss progressed substantially during treatment and he eventually required bilateral cochlear implants. His GPA relapsed towards the end of his treatment and required induction therapy with cyclophosphamide and eventually rituximab to achieve remission. Shortly after remission, he developed dyspnoea which was diagnosed to be a consequence of pulmonary fibrosis. *M. abscessus* is classified as an atypical and rapidly growing mycobacteria (RGM). It is the most common causative RGM isolated in pulmonary infections from this group of pathogens.

## Case Presentation

A 33 year old man was seen in the emergency department of our Hospital in June of 2010 with a 2 week history of fatigue and what he described as a gurgling discomfort in his chest. He was additionally coughing up blood stained sputum. He had a past medical history of GPA, diagnosed elsewhere in 2003 when he presented with progressive deafness, loss of taste, recurrent sinusitis, arthralgia and haemoptysis. He had relapsed 3 times since his initial presentation. He was on prednisone 10milligrams daily and mycophenelate motefil (MMF) 1 gram twice daily. He was also on atenolol for hypertension. He was a life-long non-smoker, but lived with both parents who were smokers.

C-reactive protein (CRP) levels were reported at < 7 grams/millilitre and erythrocyte sedimentation rate (ESR) at 32millimetres/hour. A chest x-ray showed pulmonary nodules with cavitation in both lungs. Three separate sputum cultures demonstrated *M. abscessus* infection confirmed by gene sequencing. Proteinase 3 antibody titres over the next few months remained in the negative/equivocal range 4–6 U/ml (0–5 negative, 6–9 equivocal, >9 positive). Antinuclear antibodies, double stranded DNA antibodies and extractable nuclear antibodies were all negative.

Following susceptibility testing of his mycobacterial infection and on the advice of an infectious disease specialist, he was treated with amikacin 7.5 mg/kg q12h intravenously (iv), cefoxitin 2 g q4h iv and clarythromycin 500 mg bid orally (po) for 2 months, followed by clarithromycin and amikacin to complete 12 months of antimicrobial therapy. This mycobacterium isolate was not was susceptible to oral antibiotics except clarithromycin.

He was counselled about the side effects of amickacin including deafness and consented to treatment. *Aspergillus fumigatus* was isolated from his sputum on two occasions and was treated with voriconizole. His MMF dose was slowly reduced to decrease the potential risk of immunosuppression interfering with the successful treatment of his infection.

**Figure 1:**
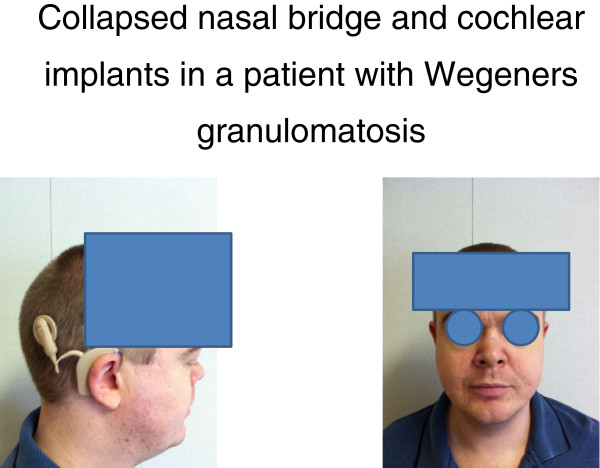
Collapsed nasal bridge and cochlear implants in a patient with GPA.

His respiratory symptoms improved until Febuary 2011 (8 months after presenting to our Unit) when he again began to feel unwell with lethargy, loss of taste, nasal crusting and discharge, senineural hearing loss, blurred vision and other symptoms. His Birmingham Vasculitis Score (BVAS) was 24 at the time [4]. Furthermore the bridge of his nose was noted to have begun to collapse (Figure 1). Repeat ANCA tests were in the range 5–6 U/ml. However his CRP now measured 108 g/ml and his ESR 75 mm/Hr. A bronchoscopy demonstrated endobronchial involvement and a transbronchial biopsy and was reported to be consistent with active GPA. For this reason, he was treated with oral cycophosphamide 1.5 mg/kg. On this occasion, he was not treated with MESNA and after 2 months of treatment, developed hemorrhagic cystitis. His treatment was then changed to rituximab 375 mg/m^2^ weekly for 4 weeks. This treatment was delayed as we sought funding for his treatment which eventually started in the middle of June 2011. Shortly thereafter, his symptoms resolved and he went into clinical remission. His PR3 levels were of 0–1 U/ml and he was kept on maintenance therapy with azathioprine.

**Figure 2:**
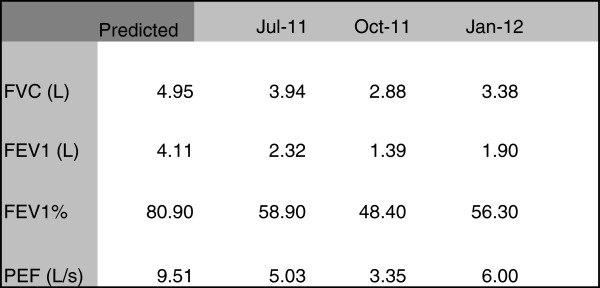
Progressive change in lung function shortly after completing treatment for GPA and Mycobacterium abscessus pulmonary infection.

**Figure 3:**
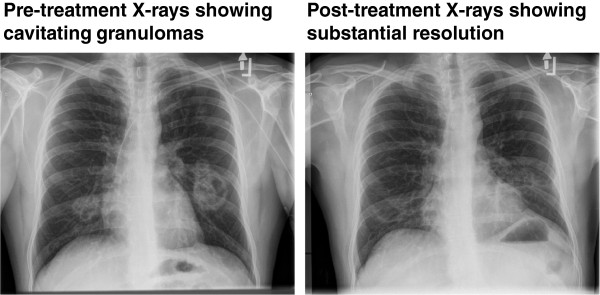
Chest radiographs demonstrating cavitation granulomas in a patient with GPA before and after treatment.

He completed his course of rituximab and antibacterial treatment in July 2011. Sputum cultures were now persistently negative and his cavitating pulmonary nodules had markedly improved, confirmed on Chest x-ray (Figure 2) and CT scan. However, shortly afterwards, he began to complain of dyspnoea. Serial spirometery showed a progressive decline in pulmonary function (Figure 3). His reduction in FEV1 was not reversible with nebulised salbutamol. Bronchoscopy was carried out in November 2011 and reported as showing evidence of widespread airways scarring particularly in the left main stem bronchus (approximately 65%) with sparing of the main trachea and carina.

During this period he developed severe mixed hearing loss in his right ear and profound sensory-neural deafness in his left ear with no measurable hearing above a frequency of 500 Hz and noise perception at 60 dB of 0%. This qualified him for cochlear implants, which were inserted in August 2011, and improved his hearing dramatically.

## Discussion

Patients with GPA are typically treated with initial immunosuppressive therapy (commonly cyclophosphamide and glucocorticoids) followed by maintenance therapy with azathioprine or methotrexate, typically for 12–18 months. However, relapses are common, occurring on average 8–9 months after ceasing immunosuppressive therapy.

Infections have been hypothesized to trigger some disease flares by inducing expression of the ANCA antigens (PR3 and MPO) on the surface of circulating neutrophils. This can, in the presence of ANCA, lead to neutrophil degranulation, the release of oxygen radicals, and vascular injury [5].

A recent study comparing cyclophosphamide with rituximab for induction therapy concluded that the rate of remission induction at six months was significantly higher with rituximab (67 versus 42%). There is however no conclusive evidence that rituximab is superior to cyclophosphamide although subgroup analysis raises the possibility that rituximab may be optimal therapy for patients with relapsing disease [6].

Pulmonary fibrosis has been rarely reported in patients with vasculitis and typically with active disease rather than after remission [7]. Additionally, substantial tissue fibrosis has been reported from kidney biopsies of patients with other ANCA associated disease [8]. ANCA antigens have a number of bioeffects and are potent activators of latent TGF-β [9] which is known to promote fibrogenesis [10]. The binding of circulating ANCA results in neutrophil degranulation and the release of reactive oxygen species which have been suggested may lead to subsequent injury and consequent fibrosis [11].

Although pulmonary fibrosis is a well-recognised complication of *M. tuberculosis* infections, we could find no case reports of this complicating *M. abscessus* lung disease. The isolation of *M. abscessus* may represent infection or colonization. However this gentleman had a short history or progressive respiratory symptoms and repeated isolation of mycobacteria from cultures which raises the likelihood of indolent disease [12]. Histological evidence to prove invasive disease may be helpful but was not done. Additionally, he was seen by our infectious diseases specialist team who felt that he likely had true infection warranting treatment.

Pulmonary fibrosis has been occasionally reported to be associated with cyclophosphamide therapy [13]. Hadjinicolau et al., [14] recently conducted a literature review to identify non-infectious pulmonary complications associated with the newer biologic agents (rituximab, certolizumab, golimumab, tocilizumab and abatacept) used for the treatment of rheumatic conditions. Interstitial lung disease, idiopathic pulmonary fibrosis, allergic pneumonitis, and culture-negative pneumonia have been reported.

Our patient developed progressive dyspnea shortly after completing induction therapy with cyclophosphamide followed by rituximab, towards the end of the treatment of his *M. abscessus* infection. Although a chest x-ray and computed tomography scans showed an improvement in the radiological appearances of his lungs, there was a marked deterioration in his FEVI, PEF and FVC suggesting both obstructive and restrictive lung disease. Bronchoscopic evaluation showed pulmonary fibrosis with arrears of significant narrowing.

Ototoxicity was an unfortunate consequence of both his GPA and prolonged treatment with amikacin. Fortunately, cochlear implants may be used in this instance and have been gaining popularity since they were first used 50 years ago for treating sensorineural hearing loss. They are surgically implanted prostheses that use electrical stimulation to provide hearing. Concerns about the ototoxicity of amikacin have led to small but successful trials of aerosolized amikacin in *M. avium intracellulare* pulmonary infections [15]. Unfortunately, the efficacy of this mode of administration in *M. abscessus* infections remains unknown.

The presence of *Aspergillus* spp. in sputum has been shown to be associated with the isolation of non-tuberculous mycobacteria in a study in involving patients with cystic fibrosis (66.7% vs. 21.5% of controls) in sputum samples [3]. Interestingly our patient had *Aspergillus* sp isolated from his sputum on two occasions during the treatment of his *M. abscessus* infection. It has been suggested that the strong association between infection with NTM and *Aspergillus* spp. may reflect the severity of the pulmonary disease or that these organisms may create favorable conditions for the co-colonization such as by altering mucociliary clearance [3].

## Conclusions

Our patient developed progressive dyspnea shortly after completing induction therapy with cyclophosphamide followed by rituximab. This coincided with the end of the treatment of his *M. abscessus* infection.

Despite a substantial improvement in the radiological appearances of his lungs, there was a marked deterioration in his pulmonary function tests and bronchoscopic evaluation demonstrated pulmonary scarring with arrears of significant narrowing.

It is difficult to be certain of the exact cause of his progressive pulmonary fibrosis. However it is plausible that his GPA, cyclophosphamide and rituximab all contributed to this because of their known potential lung toxicity. The role of *M. abscessus* if any is much less clear.

## Consent

Written informed consent was obtained from the patient for publication of this Case report and any accompanying images. A copy of the written consent is available for review by the Series Editor of this journal.

## Abbreviations

GPA: Granulomatosis with polyangitis (Wegener’s; ANCA: Anti-neutrophil cytoplasmic antibodies; PR3: Proteinase 3; RGM: Rapidly growing mycobacteria; MMF: Mycophenelate motefil; CRP: C-reactive protein; ESR: Erythrocyte sedimentation rate; DNA: Deoxyribonucleic acid; FEV1: Forced Expiratory Volume in 1 minute; PEF: Peak Expiratory Flow Rate; FVC: Forced Vital Capacity.

## Competing interest

Both authors declare that they have no competing interests.

## Authors’ contributions

AV drafted the initial manuscript and carried out the initial background literature search relating to this. He also took the photographs of the patient presented in the paper. ST was the physician with primary responsibility for the care of the patient. He suggested the use of the patient for this publication and read and corrected the initial manuscript, including a review of pertinent literature. All authors read and approved the final manuscript.

## Authors’ information

ST is a Consultant in internal medicine and nephrology who regularly manages the care of people with vasculitic illness. He trained in the United Kingdom with a basic science degree in parasitology from the University of London, followed by several years of research experience on projects funded by the World Health Organisation, prior to training in Nephrology. He then worked as Consultant at the University Hospital of North Staffordshire in England before moving to Canada. He is presently head of the Department of Medicine & Mental Health services at the Cypress Regional Hospital, Canada.

## Pre-publication history

The pre-publication history for this paper can be accessed here:

http://www.biomedcentral.com/1471-2466/12/47/prepub
